# Modulation of IL-17 and Foxp3 Expression in the Prevention of Autoimmune Arthritis in Mice

**DOI:** 10.1371/journal.pone.0010558

**Published:** 2010-05-10

**Authors:** Joana Duarte, Ana Agua-Doce, Vanessa G. Oliveira, João Eurico Fonseca, Luis Graca

**Affiliations:** 1 Instituto de Medicina Molecular, Faculdade de Medicina, University of Lisbon, Lisbon, Portugal; 2 Instituto Gulbenkian de Ciência, Oeiras, Portugal; 3 Rheumatology Department, Hospital de Santa Maria, Lisbon, Portugal; New York University, United States of America

## Abstract

**Background:**

Rheumatoid Arthritis (RA) is a chronic immune mediated disease associated with deregulation of many cell types. It has been reported that different T cell subsets have opposite effects in disease pathogenesis, in particular Th17 and Treg cells.

**Methodology and Findings:**

We investigated whether non-depleting anti-CD4 monoclonal antibodies, which have been reported as pro-tolerogenic, can lead to protection from chronic autoimmune arthritis in SKG mice – a recently described animal model of RA – by influencing the Th17/Treg balance. We found that non-depleting anti-CD4 prevented the onset of chronic autoimmune arthritis in SKG mice. Moreover, treated mice were protected from the induction of arthritis up to 60 days following anti-CD4 treatment, while remaining able to mount CD4-dependent immune responses to unrelated antigens. The antibody treatment also prevented disease progression in arthritic mice, although without leading to remission. Protection from arthritis was associated with an increased ratio of Foxp3, and decreased IL-17 producing T cells in the synovia. *In vitro* assays under Th17-polarizing conditions showed CD4-blockade prevents Th17 polarization, while favoring Foxp3 induction.

**Conclusions:**

Non-depleting anti-CD4 can therefore induce long-term protection from chronic autoimmune arthritis in SKG mice through reciprocal changes in the frequency of Treg and Th17 cells in peripheral tissues, thus shifting the balance towards immune tolerance.

## Introduction

Rheumatoid arthritis (RA) is a common chronic autoimmune inflammatory disease characterized by destruction of the synovial joints, leading to progressive disability, increased co-morbidity and premature mortality [1], [2]. Both genetic and environmental factors are known to contribute to the development of the disease [3]. RA is characterized by a complex immune mediated response with the participation of many cell types including CD4^+^ T cells [4], [5], [6], such as the IL-17 producing Th17 subset, which have been shown to play an important role in the pathogenesis of the disease [7], [8], [9]. The participation of CD4^+^ T cells in the pathogenesis of RA, namely by influencing other key cellular mediators of the disease (such as B cells or macrophages), has prompted the development of therapeutic strategies targeting this lymphocyte population [10], [11], [12], [13]. Monoclonal antibodies (MAbs) targeting key T cell molecules (such as co-receptor and co-stimulation) have been suggested as drugs capable of achieving long-term protection from the disease, with the potential of leading to immune tolerance, following a short treatment [14]. Indeed long-term transplantation tolerance can be induced in mice following CD4 or co-stimulation blockade [15], [16], [17].

The most commonly used mouse models for autoimmune arthritis – such as collagen-induced arthritis – have been instrumental in the development of new therapies, such as the blockade of key cytokines, such as TNF. However, arthritis in these mice is self-limited and, as such, pre-clinical studies of putative tolerogenic regimens aiming for long-term effects have been hampered by the lack of suitable animal models of chronic autoimmune arthritis that are not TCR transgenic.

SKG mice, harboring a mutation in ZAP-70 rendering T cells more resistant to activation and thus interfering with appropriate negative selection in the thymus, have been recently described as developing chronic autoimmune arthritis with several characteristics resembling RA [18]. Arthritis in SKG mice has a centripetal course starting with small finger joints, eventually leading to histological changes and bone destruction similar to RA [19]. The incidence and severity of the disease is greater in females, with most mice developing rheumatoid factor (RF), and some animals displaying extra-articular lesions similar to rheumatoid nodules and pneumonitis [18]. Although CD4^+^ T cells, and its Th17 subset, are important in the pathogenesis of arthritis in SKG mice, other cell populations, such as B cells, participate in the disease as suggested by the production of RF in these animals [18].

Our data reports the long-term protection from chronic autoimmune arthritis following a short course of non-depleting anti-CD4 MAb in SKG mice, associated with decreased IL-17 and increased Foxp3 expression in the synovial tissue. Furthermore, the non-depleting nature of the therapeutic MAb preserves the immune competence of treated mice.

## Methods

### Ethics Statement

All experiments involving animals were approved by the Animal User and Ethical Committees at the Instituto Gulbenkian de Ciencia, according with directives from Direccao Geral Veterinaria (PORT 1005/92). Mice were bred and maintained under specific pathogen free (SPF) conditions.

### Mice

BALB/c, DO11.10.RAG1^-/-^, and SKG mice (generously provided by Professor Shimon Sakaguchi, Kyoto, Japan). Experimental animals were between 8–10 weeks of age and sex matched.

### Autoimmune arthritis induction and anti-CD4 treatment

BALB/c and SKG mice were injected intraperitoneally (i.p.) with a single shot of 3mg curdlan per mouse. Treated mice were injected with 1 mg anti-CD4 on days 0 (the day of curdlan injection), 2 and 4. Mice treated at disease onset, were injected with 3 shots of 1 mg anti-CD4 every other day, from the day they have reached a clinical score of 0.5.

### Clinical assessment of arthritis

Joint swelling was monitored in blinded cages by two independent observers and scored as described elsewhere [18]: 0, no joint swelling; 0.1, swelling of one finger joint; 0.5, mild swelling of wrist, ankle, or base of tail; and 1.0, severe swelling of wrist, ankle or base of tail. Scores for all joints were totaled for each mouse (with a maximum score of 5 corresponding to severe swelling of the four paws and base of tail).

### Antibodies and reagents

Curdlan (Wako, Japan) and Zymosan-A (Sigma-Aldrich, USA) were dissolved in sterile PBS at 15 mg/ml and 20 mg/ml, respectively. Non-depleting anti-CD4 (YTS177) and the isotype control anti-dog CD4 (YKIX302) MAbs were produced in our laboratory using Integra CL1000 flasks (IBS, Chur, Switzerland), purified by 50% ammonium sulfate precipitation, dialyzed against PBS, and purity checked by native and SDS gel electrophoresis. The hybridomas were generously provided by Professor Herman Waldmann (Oxford, UK).

### Cytokine analysis

Evaluation of serum cytokines was performed using the mouse inflammation cytometric bead array (BD Biosciences, San Diego, CA), with beads specific for IL-6, TNF, IFN-γ, monocyte chemoattractant protein-1 (MCP-1), and IL-10. IL-17 was quantified by ELISA using a kit from R&D Systems. Cytokine concentrations were measured in duplicates, and compared with standard curves, according to manufacturer instructions.

### Quantification of rheumatoid factors

Serum levels of rheumatoid factor were measured by ELISA as previously described [18], using 5 µg/ml mouse IgG to coat the plates, and 1 µg/ml anti-mouse-IgM-HRP for detection (Southern Biotech, Birmingham, USA).

### Determination of ovalbumin-specific immune responses

On day 30 following anti-CD4 treatment, or treatment with an isotype control, the mice were immunized with two injections two weeks apart, of 20 µg ovalbumin (OVA, grade V; Sigma, St Louis, USA), in 2.0 mg of endotoxin-free aluminum hydroxide (alum, Alu-gel-S, Serva, Heidelberg, Germany), and sacrificed one week after the last immunization. Non-immunized (naïve) mice were maintained as negative controls. The serum concentration of OVA-specific immunoglobulins was determined by ELISA using an OVA-specific IgG1 kit (SouthernBiotech, Birmingham, USA) with anti-OVA IgG1 standard from Serotec (Oxford, UK).

### Histology

Ankle and metatarso-phalangical (MTP) joints were collected and cryopreserved in OCT (Sakura, NL). Cryosections were stained with hematoxilin-eosin according to standard procedures.

### Flow cytometry

Cells were stained for flow cytometric analysis with the following fluorochrome-labeled monoclonal antibodies: CD3 (145-2C11), CD4 (RM4-5), CD25 (PC61.5), and Foxp3 (FJK-16s) from eBiosciences or BD Biosciences. Intracellular cytokines were investigated in lymphocytes activated for three hours in PMA-ionomycin in the presence of Brefeldin-A. Monoclonal antibodies specific for IFN-γ (XMG1.2), IL-17A (eBio17B7), and IL-10 (JES5-16E3) were used (all from eBiosciences).

### Th17-polarization assays

OVA-specific CD4^+^ T cells from DO11.10.RAG1^-/-^ mice were purified by magnetic separation with CD4 (L3T4) microbeads (Miltenyi Biotec, Germany). Cell purities were between 92–96%. The T cells were cultured for 5 days with bone marrow derived DCs, 0.1 µM OVA peptide (New England Peptide LLC, USA), and 10 µg/ml anti-CD4 (YTS177) in IMDM 5% FBS (Invitrogen), 1% Pen/Strep (GibCo), 0.1% β-Mercaptoethanol (GibCo), 1 ng/ml TGF-β (R&D systems), 20 ng/ml IL-6 (R&D systems), 10 ng/ml IL-1β (Ebiosciences), and 10 µg/ml anti-INFγ (R46A2). At the end of the culture the cells were harvested, and processed for flow cytometry.

### RNA extraction and real time PCR

Total RNA was extracted from synovial tissue, dissected from ankle joints, using lysis buffer, and following the tissue RNA kit instructions (Omega bio-tek, USA). Foxp3 and IL-17 were quantified by real time PCR, performed on the ABI Prism® 7000 sequence detection system (Applied Biosystems, USA). The relative mRNA levels of the target genes were normalized against CD3. CD3, Foxp3 and IL-17 primers are described elsewhere [20], [21], [22].

### Statistics

Statistical significance was determined using the two-tailed non-parametric Student's t test (Mann-Whitney U). *P* values <0.05 were deemed significant.

## Results

### SKG mice develop chronic autoimmune arthritis upon systemic curdlan immunization

Although initial reports have suggested that SKG mice develop chronic autoimmune arthritis spontaneously [18], it was later confirmed that disease induction requires exposure to yeast wall extract (zymosan) or purified β-glucans, like curdlan or laminarin, acting through the pattern recognition receptor Dectin-1 [23].

**Figure 1:**
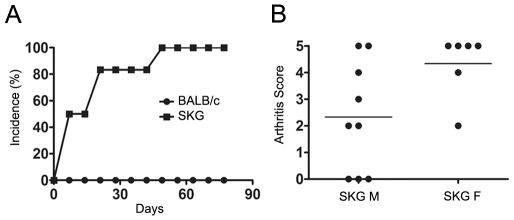
SKG mice develop chronic autoimmune arthritis upon induction with curdlan. (A) Arthritis incidence in female SKG and BALB/c mice after a single i.p. injection of 3 mg curdlan (n = 6). Curdlan was able to induce chronic arthritis in SKG but not in BALB/c mice. Each point represents the median per group. (B) Clinical score of 5 month-old male (M) and female (F) SKG mice 90 days following disease induction with curdlan. Data are from two independent experiments.

We confirmed that a single intraperitoneal injection of 2 mg Zymosan (not shown) or 3 mg Curdlan is sufficient to induce chronic disease in SKG mice, but not in wild-type controls (Figure 1A). Both male and female mice developed arthritis, although the disease was more severe in females (Figure 1B). Based on these data we decided to use curdlan in female SKG mice in subsequent experiments.

### Non-depleting anti-CD4 treatment prevents the onset of autoimmune arthritis

**Figure 2:**
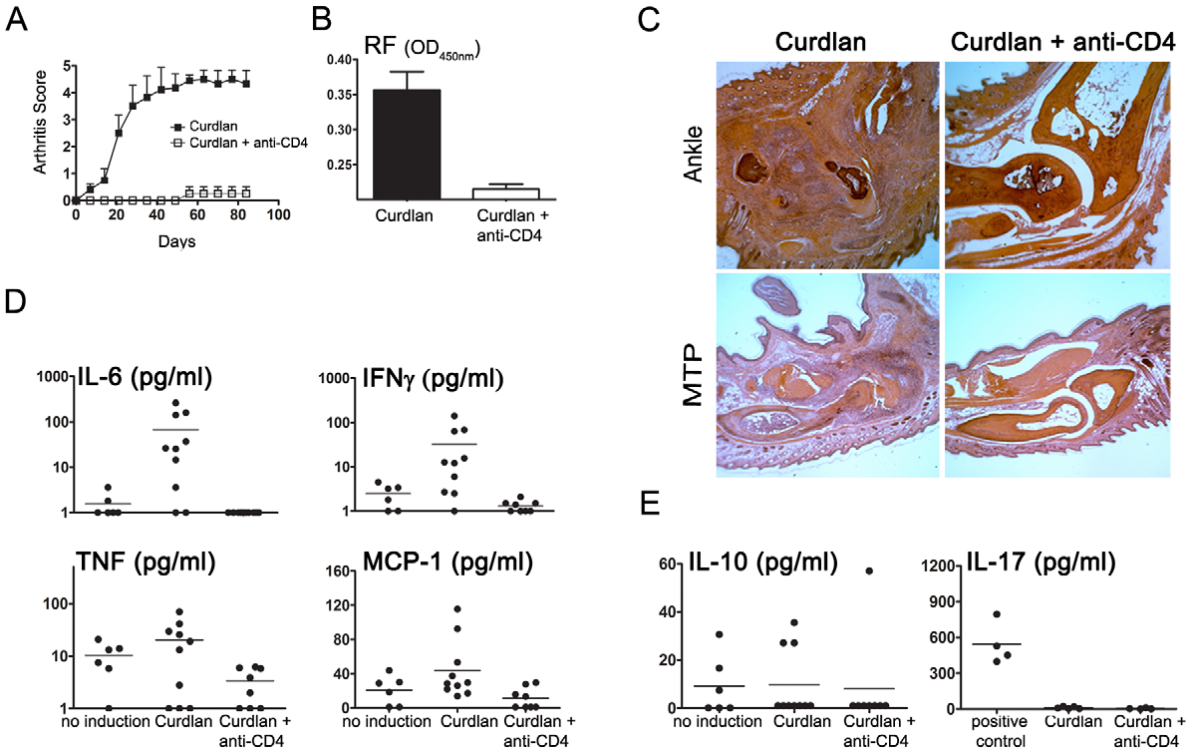
Non-depleting anti-CD4 MAb prevents the onset of autoimmune arthritis. (A) Female SKG mice were immunized with 3mg curdlan i.p. together with 1 mg non-depleting anti-CD4 or an isotype control. The MAb was administered again on days 2 and 4. Anti-CD4 treated mice were protected from the development of autoimmune arthritis (n = 6, *P*<0.001). Data, represented as mean ± SEM, are from two independent experiments. (B) Serum concentration of rheumatoid factor (RF) was measured by ELISA. Mice treated with anti-CD4 showed significantly lower levels of RF (n = 6, *P*<0.001) (C) Histological sections stained with eosin-hematoxilin from the ankle and metatarso-phalangical joints from SKG mice in the absence and in the presence of anti-CD4 treatment, 90 days following curdlan immunization. (D) Serum concentration of IL-6, IFN-γ, TNF and MCP-1 in naive SKG mice, SKG mice exposed to curdlan, or curlan and anti-CD4. Naive SKG mice were age matched and did not develop arthritis in the absence of curdlan immunization (no induction). The serum levels of IL-6 (*P*<0.05), IFN-γ (*P*<0.01) and MCP-1 (*P*<0.01) were significantly lower in anti-CD4 treated mice compared with animals injected with curldan in the absence of tolerizing MAbs. Differences in TNF concentration did not reach statistical significance. (E) The serum concentration of IL-10 and IL-17 in SKG mice exposed to curdlan, or curlan and anti-CD4 remained similar in the different experimental groups. Culture supernatants from Th17 cell culture were used as positive control.

To assess whether non-depleting anti-CD4 MAbs, suggested in previous studies as having tolerogenic potential, can lead to long-term beneficial effects in chronic autoimmune arthritis, female SKG mice were treated with non-depleting anti-CD4 together with curdlan. Anti-CD4 treatment was effective in preventing the development of autoimmune arthritis (n = 5, *P*<0.001, Figure 2A). Since arthritic SKG mice are known to produce high titres of RF (IgM anti-IgG), we quantified serum RF. We found that serum RF were below the limit of detection in all mice treated with anti-CD4 (Figure 2B).

Furthermore, animals treated with anti-CD4 not only remained without clinical manifestations of the disease, but their joints were free from inflammatory cell infiltrates. Indeed, histological sections of ankle and MTP joints from anti-CD4 treated mice showed normal joint tissues, without inflammatory infiltration or bone erosions, compared to the curdlan induced arthritic mice (Figure 2C).

We also evaluated the presence of pro-inflammatory cytokines in arthritic mice compared to anti-CD4 treated animals. We observed a significant decrease in the concentration of IL-6, TNF, MCP-1 and IFN-γ in sera from anti-CD4 treated mice, suggesting that a treatment targeting T cells can have an impact on additional cell types producing those cytokines (Figure 2D). It should be noted that IL-6, which has been associated with Th17 differentiation, is known to be critical for the pathogenesis of chronic autoimmune arthritis in SKG mice [24], and was not detectable in any of the anti-CD4 treated animals. Despite the reported association of IL-17 with arthritis in SKG mice, this cytokine was not detected in sera from arthritic animals, remaining below detection level in all groups (Figure 2E). This observation is in accordance with the proposed local effect of this inflammatory mediator.

Previous studies have suggested a protective role for IL-10 in this murine model of autoimmune arthritis [25]. However, we did not find any significant difference in the serum levels of IL-10, between arthritic and anti-CD4 treated mice (n = 10, *P*>0.05, Figure 2E).

### Non-depleting anti-CD4 alters the balance of Treg/Th17 cells in the synovial tissue and draining LNs

**Figure 3:**
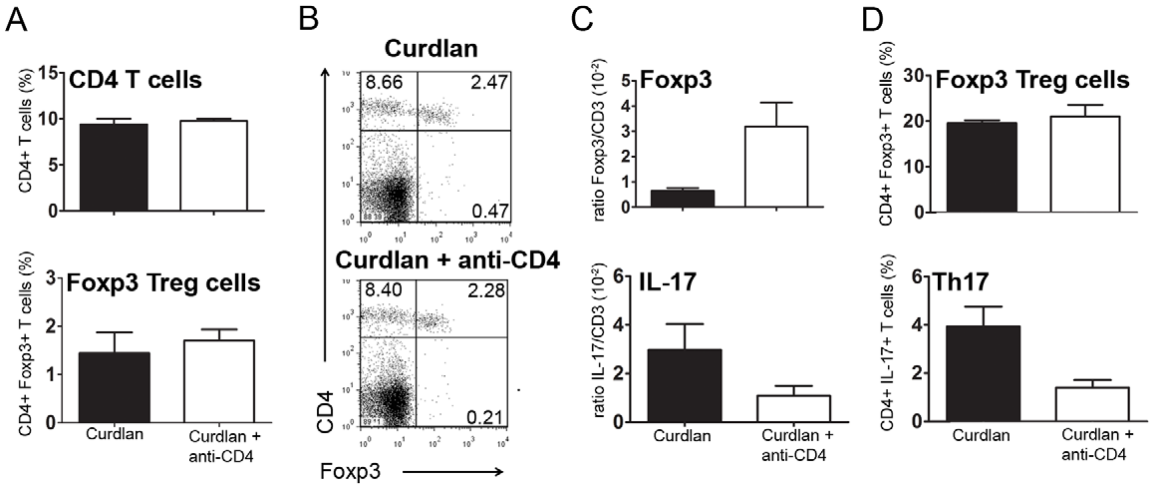
Anti-CD4 MAb influences the local balance of Th17/Treg cells. (A) Frequency of splenic CD4^+^ T cells or CD4^+^Foxp3^+^ Treg cells from SKG mice exposed to curdlan, or curdlan + anti-CD4 treatment. No significant difference was observed between the two populations of animals. (B) Representative dot plots showing the frequency of splenic CD4^+^Foxp3^+^ T cells from SKG mice exposed to curdlan, or curdlan + anti-CD4 treatment. No significant difference was observed, as represented in panel A. (C) Foxp3 and IL-17 mRNA expression from the synovial membrane of SKG mice exposed to curdlan, or curdlan + anti-CD4 (mRNA expression levels were normalized to CD3 expression). (D) Frequency of Foxp3^+^ and IL-17^+^ T cells within draining LNs of SKG mice exposed to curdlan, or curdlan + anti-CD4.

It has been reported that the anti-CD4 MAb used in our study (YTS177) has a non-depleting isotype [26]. We confirmed the non-depleting nature of the MAb as neither the splenic frequency (Figure 3A and B) nor the total number (not shown) of CD4^+^ T cells were reduced in animals treated with anti-CD4.

Transplantation tolerance achieved with non-depleting anti-CD4 has been associated with the induction of Treg cells, both in the spleen and within tolerated transplants [27], [28], [29]. We investigated if changes in Treg frequency could be seen in the spleen of anti-CD4 treated mice. We found that there was no change in T cell subpopulations of anti-CD4-treated animals, namely Foxp3^+^ Treg cells, with a similar frequency of these cells in mice exposed to curdlan in the presence or absence of anti-CD4 treatment (Figure 3A and B). In addition, the frequency of IL-17, IFN-γ or IL-10-producing T cells, identified by intracellular cytokine staining, remained constant in all groups of animals, and below 2% of the CD4^+^ T cells (not shown).

As our data show that anti-CD4 treatment can prevent joint inflammation, even though T cell subpopulations in secondary lymphoid organs appear to be unaffected, we investigated whether anti-CD4 treatment was leading to alterations in the T cell subpopulations within the synovial tissue of protected animals. Given the technical limitations for the direct enumeration of individual T cells from mouse synovial tissue, we used the expression of Foxp3 and IL-17 mRNA as surrogate markers for the relative frequency of, respectively, Tregs and Th17 cells. For this purpose Foxp3 and IL-17 mRNA expression was measured by quantitative real time PCR and normalized to CD3 expression (thus controlling for different numbers of infiltrating T cells in different samples, as the arthritic synovium contains greater numbers of T cells). This method, of using CD3 expression for normalization of different numbers of infiltrating T cells in tissues with few lymphocytes has been established for other tissues with small numbers of T cells, such as skin grafts in mice [22]. We observed that while IL-17 expression among synovial T cells (i.e. IL-17/CD3 mRNA ratio) was reduced in mice treated with anti-CD4 and consequently protected from inflammatory manifestations of the disease, the expression of Foxp3 among synovial T cells was increased in the same conditions (n = 4, *P*<0.05, Figure 3C). Thus, the Foxp3/Th17 ratio in the synovial tissue is substantially shifted following anti-CD4 treatment. In addition, the analysis of popliteal LNs, draining affected joints, showed a reduction in the frequency of IL-17^+^ T cells in anti-CD4-treated mice (n = 8, *P*<0.05) while the frequency of Foxp3^+^ cells remained similar in both groups of animals (Figure 3D).

### CD4-blockade prevents *in-vitro* Th17 polarization while favoring Foxp3 expression

Although non-depleting anti-CD4 has been widely studied for the induction of transplantation tolerance its exact mechanism of action has not been fully characterized. In experiments performed with TCR-transgenic mice devoid of Foxp3^+^ Treg cells, it was previously shown that CD4-blockade could directly lead to peripheral conversion of T cells towards Foxp3^+^ Treg cells thus achieving transplantation tolerance [22]. However, it was never assessed whether CD4-blockade could directly interfere with Th17 polarization, or whether a reduction in Th17 cells (as we observed *in vivo*) would be secondary to Treg-mediated suppression.

**Figure 4:**
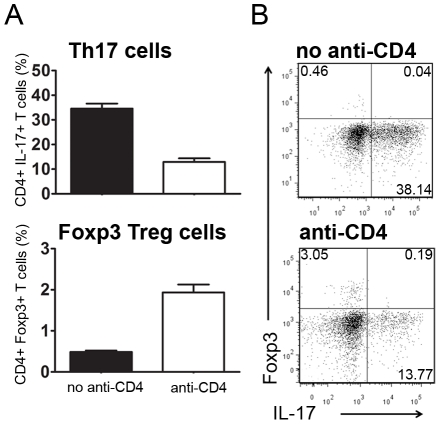
CD4-blockade prevents Th17 polarization. (A) Sorted TCR-transgenic cells were stimulated *in vitro* with peptide-loaded DCs under culture conditions known to preferentially polarize Th17 cells, with the addition of recombinant TGF-β, IL-6, IL-1β, and anti-IFN-γ. After 5 days of culture we observed a significant reduction of IL-17^+^ cells in the presence of anti-CD4 (n = 6, *P*<0.05). In contrast, anti-CD4 addition led to an increased frequency of Foxp3^+^ T cells (n = 6, *P*<0.05). (B) Representative dot plots from the two different culture conditions. An independent experiment was performed with a peptide dose of 0.3 µM with similar results.

To address this issue we investigated whether CD4-blockade could prevent *in vitro* Th17 polarization. We used OVA-specific TCR-transgenic CD4^+^ T cells sorted from DO11.10.RAG^-/-^ mice. We stimulated these cells *in vitro*, for five days, in the presence of DCs loaded with the appropriate peptide and under culture conditions promoting optimal Th17 polarization (in presence of TGF-β, IL-6, IL-1β, and anti-IFN-γ)[30]. We observed that addition of anti-CD4 led to a significant reduction of Th17 cells (n = 6, *P*<0.05, Figure 4). In spite of the presence of cytokines that promote Th17 polarization (cytokines that are known to prevent Foxp3 induction) the addition of anti-CD4 resulted in a significant increase in the frequency of Foxp3^+^ T cells (n = 6, *P*<0.05, Figure 4).

### Non-depleting anti-CD4 induces long- term protection from autoimmune arthritis

**Figure 5:**
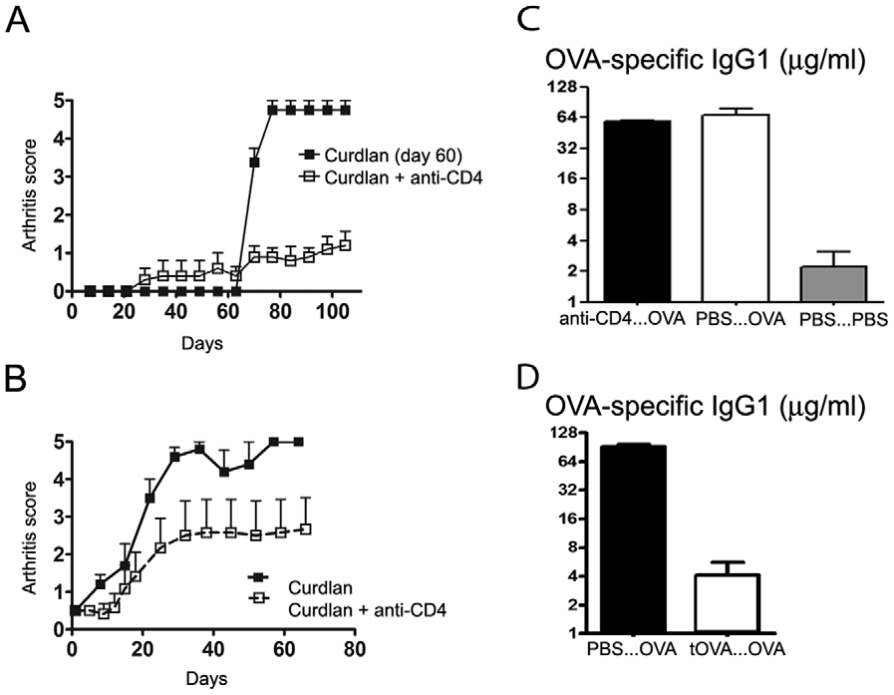
Long-term protective effect of non-depleting anti-CD4 treatment does not affect immune competence. (A) Female SKG mice were injected with 3 mg curdlan i.p. on day 0 and anti-CD4 on days 0, 2, and 4. Animals treated with anti-CD4 displayed long term protection from arthritis (n = 5, *P*<0.05). On day 60, anti-CD4 treated mice and age matched SKG were challenged with 3 mg curdlan i.p. Mice previously treated with anti-CD4 remained protected from the development of autoimmune arthritis (n = 5, *P*<0.05). (B) Female SKG mice were injected with 3 mg curdlan i.p. and, when the clinical score reached 0.5, some of the animals initiated treatment with three i.p. administrations of 1 mg anti-CD4 on alternate days. Control animals rapidly progressed to severe arthritis unlike the anti-CD4 treated mice (n = 6, *P*<0.05). (C) Mice were treated with non-depleting anti-CD4 30 days before immunization with OVA-alum i.p. (αCD4…OVA). One week following sensitization the serum levels of OVA-specific IgG1 were quantified, and compared with untreated (PBS…OVA) and non-immunized controls (PBS…PBS). Mice treated with anti-CD4 MAbs were competent to produce OVA-specific IgG1 to titres similar to untreated controls, and considerably higher than the non-immunized mice (n = 5, *P*<0.05). (D) OVA-specific IgG1 in mice where OVA was initially administered at the time of anti-CD4 treatment (tOVA…OVA), compared with animals that were not initially treated with anti-CD4 (PBS…OVA). Mice treated with anti-CD4 at the time of sensitization with OVA, became tolerant to the subsequent OVA immunization (n = 6, *P*<0.05).

To assess whether protection from arthritis induced with anti-CD4 treatment had a long-term effect, SKG mice initially injected with curdlan and non-depleting anti-CD4 were challenged with curdlan 60 days following the initial treatment. Animals exposed to curdlan in the presence of the putative tolerogenic anti-CD4 MAb at day 0 were protected from the induction of arthritis following curdlan administration at day 60, unlike the age-matched controls that did not receive any treatment at day 0 (n = 6, *P*<0.05, Figure 5A).

However, it should be noted, that some anti-CD4 treated mice developed mild manifestations of arthritis following curdlan challenge at day 60, although without progressing to the severe manifestations of the disease observed in control groups.

### Non-depleting anti-CD4 prevents progression of established autoimmune arthritis

Given the fact that non-depleting anti-CD4 treatment prevents the onset of autoimmune arthritis, we tested the effectiveness of a similar course of anti-CD4 for the treatment of established arthritis in SKG mice. Female SKG mice were immunized with curdlan and when the clinical score reached 0.5 the arthritic mice were randomly included in a group treated with anti-CD4 MAb or a control group. Mice treated with non-depleting anti-CD4 showed a long-term benefit with slower disease progression and less severe clinical scores (n = 5, *P*<0.05, Figure 5B). However, remission was only achieved in a minority of the treated animals.

### Anti-CD4 treated mice remain immunocompetent

A concern of immunomodulatory or tolerogenic therapeutic strategies is their long-term impact on the overall immune response. We therefore assessed the immune competence of anti-CD4-treated BALB/c mice (same genetic background as the SKG mice, but without the ZAP70 point mutation) to mount CD4^+^ T cell-dependent immune responses towards an unrelated antigen. For this purpose, mice treated with anti-CD4 were sensitized with 20 µg OVA-alum 30 days following anti-CD4 treatment. The quantification of OVA-specific immunoglobulins in the serum was determined one week following sensitization. Our data show that the concentration of OVA-specific immunoglobulins was similar in immunized mice, regardless of previous anti-CD4 treatment, and considerable higher than in naive controls (n = 5, *P*<0.05, Figure 5C). Moreover, we confirmed the same results in SKG mice, subjected to the same protocol (n = 2, not shown). Of note, if OVA was administered at the time of anti-CD4 treatment in BALB/c mice, the animals became unable to produce OVA-specific immunoglobulins following subsequent OVA immunization (n = 6, *P*<0,05, Figure 5D), thus proving that OVA-specific IgG production is CD4-dependent, and that tolerance is only imposed over the antigens present at the time of tolerance induction.

## Discussion

Our data show that a short course of non-depleting anti-CD4 can lead to long-term protection from the development of autoimmune arthritis, in a murine model of chronic disease. We have shown that our non-depleting antibody (clone YTS177) is not affecting T cells in the spleen, but seems to be preventing autoimmune arthritis, acting locally at the site of inflammation. It is likely that by targeting CD4^+^ T cells, the pathogenic cycle of events leading to synovial inflammation and progressive joint destruction is abrogated, as other cellular players are not recruited towards the articular tissue to cause inflammation. This impact of CD4-blockade on other cell types is well illustrated by the marked decrease of pro-inflammatory cytokines produced by dendritic cells (DCs) and macrophages in anti-CD4 treated animals. In addition, given Th17 cells have been described as involved in the production of autoantibodies in experimental autoimmune arthritis [31], [32], our observation that anti-CD4-treated mice do not produce RF is in line with the hypothesis that by maintaining pathogenic T cell clones under control the B cells will not receive the necessary stimuli for the production of autoantibodies. We cannot exclude, at this time, a direct effect of non-depleting anti-CD4 MAbs on innate cells expressing CD4, namely natural killer T (NKT) cells that have been reported as being able to provide “help” to B cells, and to influence autoimmune arthritis [33].

In our experiments, we found a limited efficacy for tolerance induction once the animals became overtly arthritic. Although we do not have a complete explanation for this observation, it is possible that such resistance to tolerance induction may be due to the participation of other cell types, besides CD4^+^ T cells, at that late time. In fact, in transplantation it is known that in pre-sensitized animals a population of Asialo GM1^+^ CD8^+^ T cells can create a barrier for tolerance induction with MAbs [34]. Thus, it may be possible to enhance the efficacy of tolerance induction in overt arthritis with reagents targeting other cell types, namely CD8^+^ T cells and B cells.

It remains to be established whether the long-term protection from arthritis afforded following anti-CD4 treatment, even after a new curdlan challenge at a later time, can be explained by the development of regulatory mechanisms that have been described in other animal models of anti-CD4 induced immune tolerance [28], [35]. In fact, it is now established that CD4^+^ T cell activation in the presence of TGF-β and IL-6 favors T cell conversion towards arthritogenic Th17 cells, while activation in the same environment devoid of IL-6 shifts the differentiation from Th17 towards Foxp3^+^ Treg cells [30]. SKG autoimmune arthritis development is known to be dependent on Th17 [36].

Our data suggests that protection from arthritis induced with anti-CD4 is associated with an overall decrease of infiltrating T cells in synovial tissue and, within those T cells in the synovium, with an increase in the frequency of synovial Foxp3^+^ Treg cells. As a consequence, the tissue is endowed with local changes that are likely to prevent the onset of arthritis directly within the local environment where inflammation would occur, even following a later exposure to curdlan at a time (day 60) where anti-CD4 MAbs are no longer present. Moreover, the reciprocal decrease of IL-17 expression at the same time that the expression of Foxp3 increases, in the joints of anti-CD4 treated mice, supports the hypothesis that indeed the balance between Treg and Th17 can determine the decision between prevention or onset of autoimmune arthritis. The observation that IL-6 decreases in the serum of anti-CD4 treated mice is also in agreement with this hypothesis. It should be noted, however, that the number of T cells is markedly reduced in the synovium of treated mice, as can be seen in the histological sections (and confirmed with greater CD3 expression in the synovia by RT-PCR – thus the need to use CD3, rather than a housekeeping gene, to normalize our gene expression studies). It was recently reported that IL-17A can be produced by mast cells in rheumatoid arthritis synovia [37]. Although we did not investigate this possible source of IL-17, the overall quantification of IL-17 transcripts was significantly reduced in anti-CD4 treated animals, where T cells were also less abundant. As a consequence, the shift in the Treg/Th17 balance in the synovial tissue is likely to be also influenced by tissue accessibility to different types of effector T cells.

We have also shown that CD4-blockade can directly inhibit T cell polarization towards an IL-17-producing Th17 phenotype, even when the most appropriate cytokine environment is provided. In addition, our data show that even in the presence of those cytokines known to inhibit Foxp3 induction (namely IL-6), CD4-blockade can facilitate the peripheral conversion of Foxp3^+^ Treg cells. Taken together, our data complements previous studies on the mechanism of tolerance induction in transplantation with anti-CD4 (where peripheral induction of Foxp3^+^ Treg cells have been shown critical), by demonstrating that CD4-blockade can also lead to a direct inhibition of Th17 polarization – a critical factor for the arthritis pathogenesis, namely in SKG mice [36].

Given the essential role of CD4^+^ T cells in the pathogenesis of RA, both directly and by recruiting and activating other participating cell types (such as B cells, DCs and macrophages), the therapeutic targeting of CD4^+^ lymphocytes has been extensively pursued [38]. Depleting and non-depleting anti-CD4 MAbs have been tested in several animal models of autoimmune arthritis [39], [40], [41], [42], and in clinical trials with RA patients [43], [44], [45]. In spite of promising results in pre-clinical studies, the therapeutic effectiveness of anti-CD4 in clinical trials was modest and short-term, possibly due to transient immunosuppression and not tolerance. In retrospect, those unimpressive results are not too surprising due to technical details related with dosing and MAb characteristics. In fact the immunogenicity of the mouse or chimeric MAb used was well documented as leading to their rapid clearance and consequent loss of efficient CD4 blockade [46]. The reduction of the number of CD4^+^ T cells was also associated with a concern of possible increased susceptibility to infection.

Our data show that non-depleting anti-CD4 can be effective in preventing chronic autoimmune arthritis while preserving immune competence, as treated mice remain able to mount a CD4-dependent immune response against a different antigen (OVA). OVA immunization is a well established protocol for the induction of CD4-dependent production of OVA-specific immunoglobulins. Moreover, administration of anti-CD4 together with OVA prevents the effectiveness of subsequent immunizations with the same antigen. Taken together, these data suggest that anti-CD4 prevents immune responses towards antigens present at the time of treatment, without hampering immune responses to unrelated antigens introduced in the organism at a later time, therefore preserving immunocompetence. It should be noted that these types of studies, on the long-term immunocompetence of treated mice have been difficult to perform in animal models that, unlike SKG mice, do not develop a chronic form of arthritis.

In summary, we show that therapeutic strategies leading to synovial accumulation of Treg cells and reduction of Th17 are capable of protecting from the onset of autoimmune arthritis, as well as to prevent long-term disease progression, without leading to overall immune suppression.
